# Chitosan-Based Hydrogels Embedded with Hyaluronic Acid Complex Nanoparticles for Controlled Delivery of Bone Morphogenetic Protein-2

**DOI:** 10.3390/pharmaceutics11050214

**Published:** 2019-05-04

**Authors:** Qing Min, Xiaofeng Yu, Jiaoyan Liu, Jiliang Wu, Ying Wan

**Affiliations:** 1 School of Pharmacy, Hubei University of Science and Technology, Xianning 437100, China; baimin0628@hbust.edu.cn; 2 College of Life Science and Technology, Huazhong University of Science and Technology, Wuhan 430074, China; m201771729@hust.edu.cn (X.Y.); liujiaoyan@hust.edu.cn (J.L.)

**Keywords:** chitosan, hyaluronic acid, nanoparticles, hydrogel, bone morphogenetic protein-2

## Abstract

Chitosan(CH)-poly(dioxanone) (CH-PDO) copolymers containing varied amounts of PDO and having free amino groups at their CH backbone were synthesized using a group protection method. The selected CH-PDO with soluble characteristics in aqueous media was used together with hyaluronic acid (HA) to prepare HA/CH-PDO polyelectrolyte complex nanoparticles (NPs) via an ionotropic gelation technique, and such a type of HA/CH-PDO NPs was employed as a carrier for delivering bone morphogenetic protein-2 (BMP-2). The optimal BMP-2-encapsulated HA/CH-PDO NPs with high encapsulation efficiency were embedded into CH/glycerophosphate composite solutions to form different hydrogels in order to achieve long-term BMP-2 release. The formulated gels were found to be injectable at room temperature and had its thermosensitive phase transition near physiological temperature and pH. They also showed abilities to administer the release of BMP-2 in approximately linear manners for a few weeks while effectively preserving the bioactivity of the encapsulated BMP-2. In view of their fully biocompatible and biodegradable components, the presently developed gel systems have promising potential for translation to the clinic use in bone repair and regeneration where the sustained and controlled stimuli from active signaling molecules and the stable biomechanical framework for housing the recruited cells are often concurrently needed.

## 1. Introduction

Local application of growth factors, such as bone morphogenetic protein-2 (BMP-2), vascular endothelial growth factor, platelet-derived growth factor, and fibroblast growth factor, alone or in combination, is recognized to be an effective strategy for bone healing because their systemic administration is impractical due to their short in vivo lifetime [1,2]. Among the mentioned factors, BMP-2 is highly potent for stimulating bone formation at the applied sites, which makes it an alternative for autogenous bone healing without the need for graft harvest. BMP-2 has now been clinically used for the treatment of bone fractures and in spinal fusion procedures [2,3]. Nevertheless, the administration of BMP-2 in the form of solutions often does not yield the desired outcomes in bone healing due to its rapid diffusion from the site of administration. A suitable carrier is usually needed to prolong its retention at the applied site with proper dosage and sustained activity maintenance if BMP-2 based treatments are intended for bone repair or regeneration [3,4].

Various kinds of biodegradable natural polymers have been adapted for use in local delivery of BMP-2, and collagen-based materials are found to be effective carriers for the administration of BMP-2 [4,5]. Nowadays, a type of bovine absorbable collagen sponge (ACS) is being used in a clinical setting for delivering BMP-2 [5]. Despite many successful cases associated with the ACS carrier, it has some intrinsic shortcomings, such as the high initial burst release and consequent inability to provide long-term BMP-2 release, which could result in inferior outcomes in bone repair or regeneration while exposing patients to the risk of various side effects [2,6]. Since very few carriers are currently available for the BMP-2 clinical use, efficacious delivery systems with clinical translation potency are still needed to be explored for localizing BMP-2 at the defect site and controlling its release at an appropriate dose over a sufficient period of time. 

In the case of bone repair, injectable hydrogels have attracted a lot of attention because they are able to form into solid-like objects in situ at the bone defects via minimal invasive injection procedures, and conveniently fill complex defects [7]. In addition, they can also act as carriers for delivering cells, therapeutic drugs and bioactive molecules [7,8]. To date, many hydrogels based on natural polymers have been used in bone healing and regeneration [8]. Among them, chitosan (CH)/glycerophosphate (GP) hydrogel has received strong interest due to its thermogelling nature near physiological temperature and pH [9], and it has already been used for repairing various types of tissue defects [10]. Although CH/GP gel has the potency to serve as an injectable carrier for delivering growth factors, the direct incorporation of growth factors within CH/GP gel would result in burst release of the loaded factor due to porous features and high water content of the gel, which is disadvantageous to the factor-involved tissue repair [10].

In this study, an effort was made to embed BMP-2-encapsulated nanoparticles into the CH/GP gel to examine whether the resulting gel system is able to sustain the release of BMP-2 in a controlled manner for a sufficient period of time. A series of chitosan-poly(dioxanone) (CH-PDO) copolymers was synthesized by grafting PDO side chains onto the C-6 sites of CH units while leaving the amino groups at the CH backbone free. The selected CH-PDO with soluble character in aqueous media was used together with hyaluronic acid (HA), a negatively charged natural polysaccharide, to produce BMP-2-encapsulated HA/CH-PDO complex nanoparticles (NPs). The BMP-2-encapsulated HA/CH-PDO NPs with optimized surface electrical property were embedded into the CH/GP gel to build a new type of gel for the release administration of BMP-2. Some formulated gels were found to have injectable and thermosensitive properties, and furthermore, to show affirmative abilities to administer the release of BMP-2 in well-controlled manners for a few weeks while effectively preserving the bioactivity of the released BMP-2. Since these gels are completely composed of biocompatible and biodegradable components, they could act as injectable scaffolds with certain functions similar to the extracellular matrix (ECM) for housing cells besides their capacity for administering the BMP-2 release. Some results related to the preparation and characterization of the gel system and to the BMP-2 release patterns as well as its bioactivity evaluation were reported.

## 2. Materials and Methods

### 2.1. Materials

CH was procured from Aladdin Inc (Shanghai, China). Viscosity average molecular weight and deacetylation degree of CH were determined as 1.19 (±0.15) × 10^5^ and 92.6 (±1.8)% using reported methods [11,12]. Human recombinant BMP-2 and BMP-2 ELISA Kit were purchased from PeproTech Inc (Rocky Hill, NJ, USA) and Abcan Inc (Shanghai, China), respectively. HA (sodium salt, Mw: 90–110 kDa), GP and sodium tripolyphosphate (TPP) were purchased from Sigma-Aldrich (Shanghai, China). All other reagents and chemicals were of analytical grade and purchased from Sinopharm (Shanghai, China).

CH-PDO copolymers were synthesized by grafting PDO side chains onto the C-6 sites of CH backbone using a group-protection method described elsewhere [13], and the optimized CH-PDO with a PDO weight percentage of about 30 wt% was used for the preparation of nanoparticles.

### 2.2. Preparation of Nanoparticles

Blank HA/CH-PDO NPs and HA/CH NPs were first prepared to optimize their compositions and properties and also used for examining the rheological properties of NP-embedded hydrogels. These NPs were prepared following a method similar to that described elsewhere [14]. In a typical preparation process, the selected CH-PDO was dissolved in 1.0% acetic acid to prepare a solution with a CH-PDO concentration of 0.6 mg/mL; and a 1.2 mg/mL HA solution was also prepared by dissolving HA in deionized water. TPP solution (150 μL, 0.75 mg/mL) was introduced into 1 mL of HA solution and the resulting HA/TPP solution was mixed with the CH-PDO solution at various HA/CH-PDO mass ratios of 4:2, 4:3 and 4:4 at room temperature. After that, magnetic stirring was maintained for 20 min for the complete formation and stabilization of NPs. The obtained blank HA/CH-PDO NPs were isolated by centrifugation, and the collected NPs were lyophilized for further use. The blank HA/CH NPs were also prepared with the same method. 

BMP-2 encapsulated HA/CH-PDO NPs were produced using a similar method. In brief, varied amounts of BMP-2 were introduced into the CH-PDO solution with stirring, and the BMP-2 contained CH-PDO solutions were mixed with the above prepared HA/TPP solution at the same HA/CH-PDO mass ratios of 4:2, 4:3 and 4:4, respectively. The BMP-2 encapsulated HA/CH-PDO NPs were retrieved by centrifugation. BMP-2 encapsulated HA/CH NPs were also prepared and used as control. All of these kinds of NPs were prepared under the same processing conditions, using a fixed amount of TPP, only changing the composition ratio. Relevant parameters for these NPs were presented in Table 1 and Table 2, respectively. The BMP-2 content in NPs was determined using BMP-2 ELISA Kit. Encapsulation efficiency (EE) of NPs was calculated by the following equation:

EE(%) = (M_0_/M_1_) × 100% (1)

where M_0_ is the mass of the BMP-2 inside NPs, and M_1_ is the mass of the fed BMP-2.

### 2.3. Characterization 

^1^H NMR measurements were conducted on a spectrometer (BrukerAV500, Rheinstetten, Germany) using DMSO-*d_6_* as a solvent. The PDO percentage in CH-PDOs was determined by elemental analysis measurement (Vario EL III, Elementar, Hanau, Germany). Fourier transform infrared (FTIR) spectra of different samples were performed on a spectrometer (VERTEX 70, Bruker, Ettlingen, Germany). The morphology of NPs was viewed using a transmission electron microscope (TEM, Tecnai G2, FEI, Hillsboro, OR, USA) and scanning electron microscope (SEM, Quanta 200, FEI, Eindhoven, Netherlands). Hydrodynamic size and zeta (ζ) potential of NPs were measured using a dynamic light scattering instrument (Nano-ZS90, Malvern Instruments, Worcestershire, UK). Weighed dry NPs (W_d_) were immersed in a phosphate buffer saline (PBS, pH:7.2) solution at 37 °C for 2 h. They were then transferred into some thimbles and the excess water was removed by centrifugation at 2000 rpm for 1 min. Weight (W_s_) of swollen NPs was measured and their swelling index (SI) was calculated as follows:

SI = [(W_s_ − W_d_)/W_d_] × 100%. (2)

### 2.4. Preparation of Composite Solutions

A series of composite solutions without BMP-2 loading was prepared using CH, GP, blank HA/CH NPs and blank HA/CH-PDO NPs in various proportional compositions, and these solutions were used for evaluating the rheological properties of resulting hydrogels. Relevant data for compositions of NP-embedded CH/GP gels are summarized in Table 3. On the basis of proportions listed in Table 3, different BMP-2-encapsulated composite solutions were prepared by embedding the optimal BMP-2-encapsulated NPs into the CH/GP gel for subsequent assessment, and the formulations for them are presented in Table 4.

Gelation time was estimated using the inverted tube testing method. Briefly, one of the prepared composite solutions (1.5–2.0 mL) was stirred in an ice/water bath for around 5 min before being introduced into a glass vial and the vial was then incubated in a water bath maintained at 37 °C. The solution flowability was examined by inverting the vial, and gelation time was recorded starting from the incubation of the vial and ending at the moment when the solution stopped flowing. 

### 2.5. Rheological Measurement 

Rheological measurements of composite solutions or hydrogels were conducted on a rheometer (Kinexus Pro KNX2100, UK) equipped with a parallel-plate sample holder. The storage modulus (G′) and loss modulus (G″) of samples were measured at a constant strain amplitude of 1% and a frequency of 1.0 Hz. Temperature dependence spectra of G′ and G″ were recorded in a temperature ranging from 25 to 45 °C at a temperature-elevated rate of 1 °C/min, and the incipient gelling temperature (T_i_) of composite solutions was determined at the intersection point of G′ and G″. 

### 2.6. In Vitro Release of BMP-2 

CH/GP solution (0.5 mL) containing a prescribed amount of BMP-2-encapsulated NPs (see Table 4) was filled into a cylindrical mold (diameter: 10 mm) and the mold was incubated at 37 °C for 20 min to allow for the gel formation. The obtained gel samples were introduced into vials, followed by addition of 4 mL of PBS. The vials were vortexed on a shaking table at 37 °C and 60 rpm. At predetermined time intervals, 1 mL of medium was withdrawn while replenishing with the same volume of fresh buffer. The released amount of BMP-2 was measured using BMP-2 ELISA Kit. 

For comparison, BMP-2 encapsulated NPs were also measured to determine their release profiles. In brief, weighed amounts of BMP-2 encapsulated NPs (10 mg) were introduced into glass vials filled with 3 mL PBS. These vials were agitated on a shaker (60 rpm) at 37 °C. At each time point, the supernatant of each specimen was withdrawn and replenished with the same volume of fresh buffer. BMP-2 concentrations were determined using BMP-2 ELISA kit.

### 2.7. Bioactivity Assessment of Released BMP-2 

The bioactivity of the released BMP-2 was assessed by evaluating BMP-2’s ability to induce alkaline phosphatase (ALP) activity in C2C12 cells [15,16]. C2C12 cells (Sixin Biological Technology Inc., Shanghai, China) were seeded in 24-well culture plates (5 × 10^4^ cells/well) with DMEM containing 2% fetal bovine serum (Thermo Fisher Scientific, Waltham, MA, USA) and 1% penicillin/streptomycin (Sinopharm Inc, Shanghai, China) and incubated at 37 °C in a 5% CO_2_ atmosphere for 6 h. After that, cells were exposed to the released BMP-2 and incubated under standard conditions for various periods up to 7 days with culture medium change every two days. The applied BMP-2 amount was set as 10, 20 and 40 ng/mL, respectively. Cells responded to free BMP-2 at the matched equivalent BMP-2 doses were used as control. BMP-2-stimulated C2C12 cells were lysed by exposing them to 0.5% Triton X-100 in PBS, followed by frozen-thaw treatments. The obtained lysates were centrifuged at 12,000 rpm at 4 °C for 10 min. ALP activity in the supernatants was measured photometrically at 405 nm using an ALP detection Kit (Beyotime, Shanghai, China) basing on the reduction of p-nitrophenyl phosphate to p-nitrophenol [15,16]. 

### 2.8. Statistical Analysis 

Data were presented as mean ± standard deviation. Analysis of the difference between groups was performed using one-way ANOVA. The statistical difference was considered to be significant when the *p*-value was less than 0.05. 

## 3. Results and Discussions 

### 3.1. Parameters of Nanoparticles 

CH is a natural cationic polysaccharide and it has been widely used in different biomedical areas due to many of its advantages related to biodegradability, hydrophilicity, non-toxic and non-antigenic properties, anti-microbial activity, bioadherence and cell affinity as well as hemostatic potency [17,18,19]. CH NPs are often functioned as carriers for delivering a variety of drugs or protein factors [19,20]. Despite these, CH NPs usually are not suitable for the long-term administration of protein factor owing to their initial burst release characteristics even though varied kinds of crosslinkers have been applied [19,20]. Poly(dioxanone) (PDO) is a kind of biodegradable and biocompatible polyester with mechanically strong and tough features [21]. It can be nearly completely depolymerized under conditions of reduced pressure and suitable temperatures, enabling it to be an environment-friendly polymer [21,22]. By grafting PDO oligomers onto chitosan units, the resulting CH-PDO could be soluble in aqueous media while showing the merits of both CH and PDO [13]. In the present study, a series of CH-PDOs with varied substitution degrees of PDO was synthesized by grafting PDO onto the C-6 sites of CH backbone while leaving the amino groups at the C-2 sites of CH units free for subsequent crosslinking. Figure S1 provides FTIR spectra for chitosan, PDO and CH-PDO; and a representative ^1^H NMR spectrum for the obtained CH-PDO. It confirms that the PDO side chains have been successfully conjugated onto the CH backbone [13,22]. Among the obtained CH-PDOs, the CH-PDO with a PDO content of about 30 wt% was selected for the follow-up preparation of complex NPs, taking into consideration the solubility of the CH-PDO in the 1.0% aqueous acetic acid solution. 

The amino groups of CH units can be crosslinked by different covalent crosslinkers such as genipin, glutaraldehyde, diisocyanate, ethylene glycol diglycidyl ether, glycidoxypropyltrimethoxy silane or some ionic crosslinkers, including calcium sulfate, sodium citrate and sodium tripolyphosphate (TPP) [19]. Among these crosslinkers, genipin and TPP have been considered to be relatively safe for in vivo use [19,20]. TPP has been very often used to crosslink CH NPs that are intended for the delivery of bioactive molecules because it is able to interact with the protonated amino groups of CH via electrostatic forces without impairing the bioactivity of the loaded molecules. Taking into account the similarity between CH and CH-PDO in their free amino groups, in the present study, TPP was selected for crosslinking CH-PDO NPs. Several studies show that TPP-crosslinked CH NPs usually carry a certain amount of surface positive charges, indicated by high positive ζ potential [23,24]. Our tentative experimental results also exhibited that TPP-crosslinked CH-PDO NPs had high positive ζ potential, and such surface properties make them unsuitable for being embedded into CH/GP gel because the resulting gels showed pronouncedly elevated gelling temperature and considerably prolonged gelling time. 

**Figure 1:**
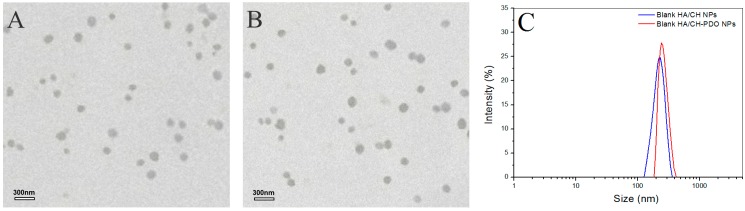
Transmission electron microscope (TEM) images of blank HA/CH NPs (hyaluronic acid/chitosan nanoparticles) (**A**) and blank HA/CH-PDO NPs (hyaluronic acid/chitosan-poly(dioxanone) nanoparticles) (**B**) as well as their size distribution (**C**).

In the light of anionic features of HA, it could be feasible to combine CH-PDO with HA to form TPP-crosslinked HA/CH-PDO complex NPs with certain electrical surface properties so that they might not significantly influence the gelling temperature and time once being embedded into CH/GP gel. In a previous study, one type of HA/CH complex NPs was developed using TPP as a crosslinker, and their ζ potential was found to be altered within a large positive and negative potential interval [14]. The similar method was adopted in the present study. An effort was made to achieve TPP-crosslinked HA/CH-PDO complex NPs with approximately electrical neutral surface property in order to enable them to be embedded into the CH/GP gel without disturbing the gelling temperature and time of the gel itself. Several sets of blank HA/CH-PDO NPs and their partners, blank HA/CH NPs, were first prepared for optimizing the composition, size and electrical surface property of these NPs, and relevant results and parameters are presented in Figure 1, Figure S2 and Table 1.

**Figure 2:**
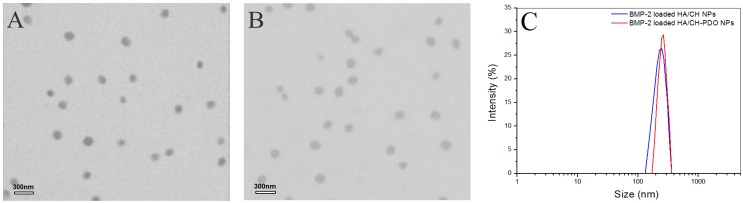
TEM images of BMP-2-encapsulated HA/CH NPs (**A**) and BMP-2-encapsulated HA/CH-PDO NPs (**B**) as well as their size distribution (**C**).

Figure 1 shows representative TEM images and size-distribution for different blank complex NPs, and their matched SEM images are displayed in Figure S2. These blank NPs were approximately spherical and some of them had the wrinkled surface (see Figure S2). Their hydrated size showed an approximate Gaussian distribution and varied from more than 100 to around 400 nm (Figure 1C). Two kinds of blank NPs looked similar in morphology and size range. Data in Table 1 indicated that for two types of NPs, their mean size increased with increasing HA content in the NPs, and concomitantly, ζ potentials changed from positive to negative values, with an absolute difference reaching to around 50. Among them, AN2 and AN5 had similar size, and importantly, their surface was approximately electrical neutral in view of their small negative ζ potential, denoting that they could be suitable for embedding into the CH/GP gel without significantly modifying the gelling temperature and time of the resulting gels. All kinds of NPs had large SI without significant differences (*p* > 0.05) among them. The high swelling rate of these NPs can be ascribed to their high content of water-soluble HA component. Based on the results shown in Table 1, BMP-2-encapsulated HA/CH-PDO complex NPs were prepared by keeping the mass ratio of HA to CH-PDO at 4:3 to achieve necessitated NPs with their size and electrical surface property similar to their respective blank NP counterparts. Figure 2 exhibits two typical TEM images and size-distribution for BMP-2-encapsulated NPs, and the matched SEM images are presented in Figure S3. These images signify that both kinds of BMP-2-encapsulated NPs had similar morphology (see Figure S3). When compared to Figure 1 and Figure S2, it can be noted that these BMP-2-encapsulated NPs looked similar to their respective blank NP counterparts in morphology and size. A few sets of BMP-2-encapsulated NPs were prepared using various amounts of BMP-2 under the same processing conditions, and relevant parameters for them are listed in Table 2. Data in Table 2 point out there were no significant differences in size and complex NPs, suggesting that addition of BMP-2 into these complex NPs has not exerted significant impacts on their mean size and ζ potential. These results are rational since the mass of encapsulated BMP-2 in these NPs is very low in comparison to the mass of the NP matrix. It can be seen from Table 2 that three kinds of BMP-2-encapsulated HA/CH-PDO NPs showed significantly higher EE than that for their respective corresponding BMP-2-encapsulated HA/CH NPs. The differences between them could be ascribed to the different interactions that exist in NPs. There are several interactions in these complex NPs, majorly including physical entanglements of different molecular chains, TPP-associated ionic crosslinking and electrostatic force between amino groups of CH chains and carboxyl groups of HA chains. Despite the similarity between two types of NPs, the employed CH-PDO component in BN4, BN5 and BN6 NPs has hydrophobic PDO side chains, and these PDO branches could function as hooks to drag the BMP-2, HA and CH main chains together, contributing additional binding force to hold a larger amount of BMP-2 molecules inside these NPs than that in BN1, BN2 and BN3 NPs, reflected by their higher EE. Table 2 also elucidates that the BMP-2 feed amount imposed certain effects on EE of BMP-2-encapsulated HA/CH-PDO NPs. BN5 NPs had their EE significantly higher (*p* < 0.05) than that for BN4, but there was no significant difference (*p* > 0.05) in EE between BN5 and BN6. Similarly, these BMP-2-encapsulated NPs also had large SI and the ratio of HA to CH or CH-PDO appeared to function as a key factor for regulating SI of the NPs. High EE is a key issue that is associated with rational use of BMP-2 due to the high cost of BMP-2, and thus, BN5 NPs were selected to be incorporated in the CH/GP gel for administering the release of BMP-2 whereas their counterpart, BN2 NPs, was used as control.

### 3.2. Gelation Properties of Hydrogels 

**Figure 3:**
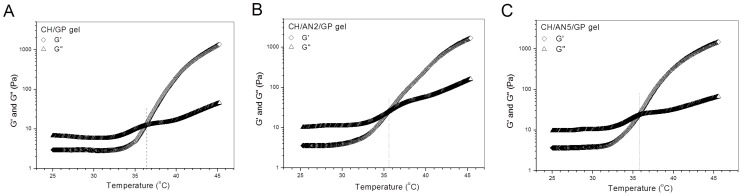
Typical temperature-dependence transitions of G′ and G″ for three kinds of gels without BMP-2 load (CH in these gels: 2.0 *w*/*v*%; GP content in these gels: 5.0 *w*/*v*%; AN2 NPs: 1.5 *w*/*v*%; and AN5 NPs: 1.5 *w*/*v*%; see Table 1 for parameters of AN2 and AN5 NPs).

**Figure 4:**
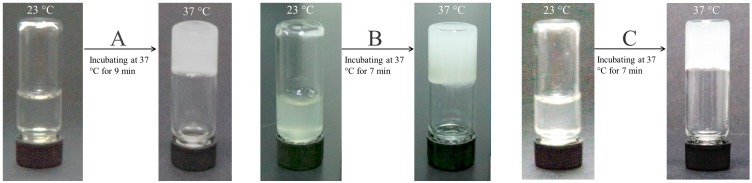
Photos for the sol-gel transition of CH/GP gel (**A**), CH/AN2/GP gel (**B**) and CH/AN5/GP gel (**C**) incubated at 37 °C for varied periods of times (see Figure 3 for the composition of these gels).

Data in Table 1 and Table 2 explicate that BN2 and BN5 NPs are respectively similar to their blank NP counterparts, namely, AN2 and AN5 NPs, in size and ζ potential. Therefore, AN2 and AN5 NPs can be used to take the place of BN2 and BN5 NPs for preparing NP-embedded gels without BMP-2 loading and for evaluating the gelation properties of the resulting gels. By doing so, costly BMP-2 can be saved. Figure 3 elucidates the several temperature-dependence curves of G′ and G″ for three kinds of gels. CH/GP gel had its T_i_ at around 36 °C, and two others, namely, CH/AN2/GP and CH/AN5/GP gels, showed their T_i_ slightly lower than 36 °C, meaning that incorporation of these complex NPs into the CH/GP gel does not significantly alter the gelling temperature of the CH/GP gel itself. Several photos for the sol-gel transition of different composite solutions incubated at 37 °C are displayed in Figure 4. The photos show that (i) three kinds of solutions behaved like a fluid at room temperature and they were able to transform into gels at 37 °C; (ii) gelling time for the NP-embedded gels was shorter than that of the CH/GP gel. Several sets of gels containing AN2 or An5 NPs were measured for their pH and T_i_ as well as gelling time, and relevant parameters are presented in Table 3. Results enumerated in Table 3 reveal that (i) all gels showed their pH value at around 7; and (ii) NP-embedded gels had their T_i_ very similar to that for the CH/GP gel without significant differences (*p* > 0.05). These results suggest that NP-embedded composite solutions are able to form into gels near physiological temperature and pH, and the amount of the embedded NPs does not significantly alter T_i_ of the gels. On the other hand, it can also be seen from Table 3 that gelling time at 37 °C for the NP-embedded gels significantly changed as the amount of the embedded NPs increased.

In principle, a too long or short gelling time is disadvantageous to the in vivo application of injectable gels. In general, an injectable solution with gelation properties needs to be fast shaped into the desired solid-like object with suitable strength and stability because otherwise the ungelled portion of the solution could transude from the injection site to form into small gelatinous beads and randomly migrate from one site to another, leading to some unwanted effects [7,25]. However, on the other hand, in some gel applications, for example, for delivering cells, it will take time to prepare the cell-loaded injectable mixtures at 37 °C, thereby requiring the resulting mixtures to remain well-defined flowability and injectability over a certain period of time before their gelling [7,8,26]. The gelling time of the CH/GP gel is suitable for many different situations as it has been extensively investigated for a variety of biomedical applications [7,10,27]. On the current situation, among these NP-embedded gels, the minimum gelling time is about 7 min, which is only 2 min less than that for the CH/GP gel, suggesting that they should be suitable for injection applications in term of their gelling time. On the basis of the results illustrated in Table 3, the NP-embedded gels with BMP-2 loading were thus prepared following the formulations shown in Table 3. Relevant parameters for BMP-2 encapsulated gels are provided in Table 4. In comparison to Table 3, it can be noticed that these gels had their respective gelling time and T_i_ very similar to the corresponding ones elucidated in Table 3, confirming that the presently employed methodology involving the utilization of blank NPs in place of BMP-2 encapsulated NPs for gel preparation is rational and effective. 

### 3.3. In Vitro BMP-2 Release

**Figure 5:**
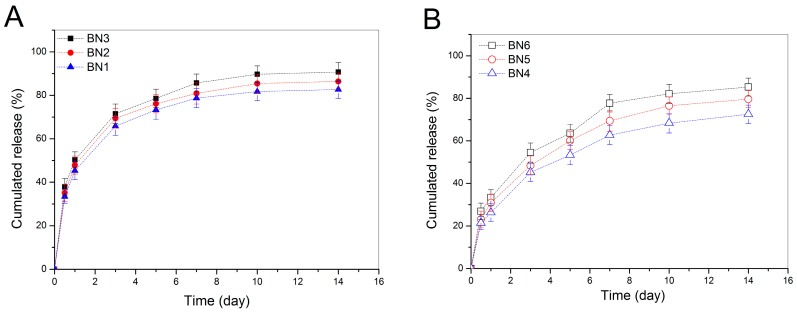
Release profiles for BMP-2-encapsulated HA/CH NPs (**A**) and for BMP-2-encapsulated HA/CH-PDO NPs (**B**) (initial BMP-2 load for BN1, BN2, BN3, BN4, BN5, and BN6 NPs was 13.3, 30.8, 48.5, 19.1, 41.9 and 63.5 ng/mg, respectively; see Table 2 for their other parameters).

BMP-2-encapsulated NPs were tested to see their BMP-2 release behavior and the release profiles as a function of time are illustrated in Figure 5. It can be seen that BN1, BN2 and BN3 NPs showed quite a similar release pattern with dramatically initial burst release, and around 50% of the incorporated BMP-2 was released from them on the first day (Figure 5A). The cumulative BMP-2 release reached about 70% for these NPs after 3-day release. It is also observed that the initial BMP-2 load seemed not to have any substantial impacts on their release profiles. In cases of BN4, BN5 and BN6 NPs, they also had similar release patterns with significantly reduced initial release rate when respectively compared to BN1, BN2 and BN3 NPs in order. Release patterns of these NPs reached a plateau region after 10-day release, and more than 60% of the incorporated BMP-2 was released by day 14. These results are reasonable if more details for them are figured out. As described in the experimental section, BMP-2 was physically encapsulated into HA/CH NPs. BMP-2 molecules could thus entangle other components in the NPs or be just simply trapped by the TPP-crosslinked chain network inside the NPs. Once these NPs were exposed to PBS media, they would be remarkably swelled because HA component in the NPs is water-soluble, TPP-induced ionic linkages are loose in aqueous media, and meanwhile, CH component is highly hydrophilic. The highly swollen BMP-2-encapsulated HA/CH NPs would allow BMP-2 molecules to disentangle and to easily diffuse into the surrounding media, leading to their fast release. With respect to BMP-2-encapsulated HA/CH-PDO NPs, namely, BN4, BN5 and BN6 NPs (see Table 2), many BMP-2 molecules in the NPs could be strongly dragged by the PDO branches in the CH-PDO main chains and are hard to be released because the PDO branches are hydrophobic and could act as hooks to drag BMP-2 molecules. As a result, BMP-2 release from these NPs would be significantly decreased in the initial period and be notably slowed down in the later stage as compared to that from BMP-2-encapsulated HA/CH NPs (Figure 5B). The results shown in Figure 5 indicate that all these NPs have severe initial burst release characteristics and fast release rates, suggesting that they are not capable of administering the long-term release of BMP-2.

**Figure 6:**
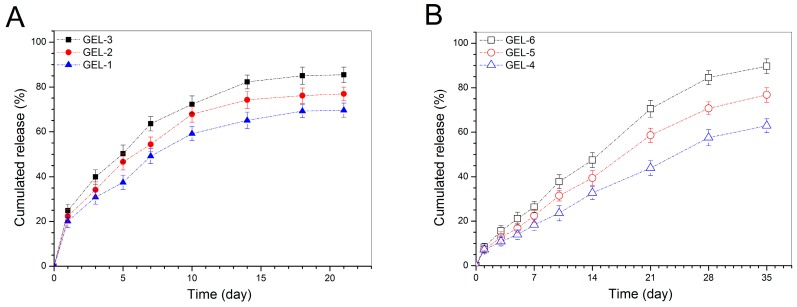
Cumulative BMP-2 release profiles for the gels embedded with BMP-2-encapsulated HA/CH NPs (**A**) and with BMP-2-encapsulated HA/CH-PDO NPs (**B**).

Figure 6 exhibits release profiles of BMP-2 for the gels embedded with BMP-2-encapsulated NPs. Curves in Figure 6A indicate that three kinds of gels had a fast release rate on the first day with a released amount of around 20% or higher. After that, the BMP-2 release became slower with varied rates somewhat depending on the initial loading amount of BMP-2 in the gels. In contrast to observations related to BMP-2-encapsulated NPs illustrated in Figure 5, these gels had significantly reduced initial burst release, and meanwhile, from the end of day one, they showed abilities to administer the BMP-2 release for a few weeks. The significantly reduced initial and subsequent release rates for these gels can be partially attributed to the contribution of the gel matrix. As denoted in Table 4, NP-embedded gels were prepared by dispersing BMP-2-encapsulated NPs into the CH/GP gel. Under these circumstances, BMP-2 molecules need to first diffuse out of the NPs and then to transport through the gel matrix to reach the release medium. On this journey, BMP-2 molecules will face resistance arisen from both NPs and gel matrix, which will certainly slow down the release rate of BMP-2 when compared to that matching with BMP-2-encapsulated NPs themselves.

Figure 6B points out that BMP-2 was released from GEL-4, GEL-5 and GEL-6 gels in quite a different way in comparison to that shown in Figure 6A. The initial burst release from these gels was largely reduced and the BMP-2 release on the first day was recorded to be less than 10%. These gels showed capacities in controlling the release of BMP-2 in approximately linear manners for a few weeks, and their BMP-2 release rate appeared to be related to their BMP-2 loading after one week of release. As shown in Table 4, the key difference between GEL-*m* (*m* = 1, 2 and 3) gel set and GEL-*n* (*n* = 4, 5 and 6) gel set is that two types of NPs were respectively used to prepare these two sets of gels. Accordingly, the approximately linear release behaviors for GEL-4, GEL-5 and GEL-6 gels should be associated with the synergistically controlled effects of the CH-PDO component in the BMP-2-encapsulated HA/CH-PDO NPs and the gel matrix. As for the differences in the BMP-2 release rate of these gels at later release stages, the possible reason is that among them, the gel containing a higher amount of BMP-2-encapsulated HA/CH-PDO NPs would have a higher BMP-2 concentration inside the gel during the release period, which will allow BMP-2 molecules to faster diffuse through the gel and get into the media due to the presence of concentration gradient, resulting in higher cumulative BMP-2 release.

### 3.4. Bioactivity Assessment of Released BMP-2

**Figure 7:**
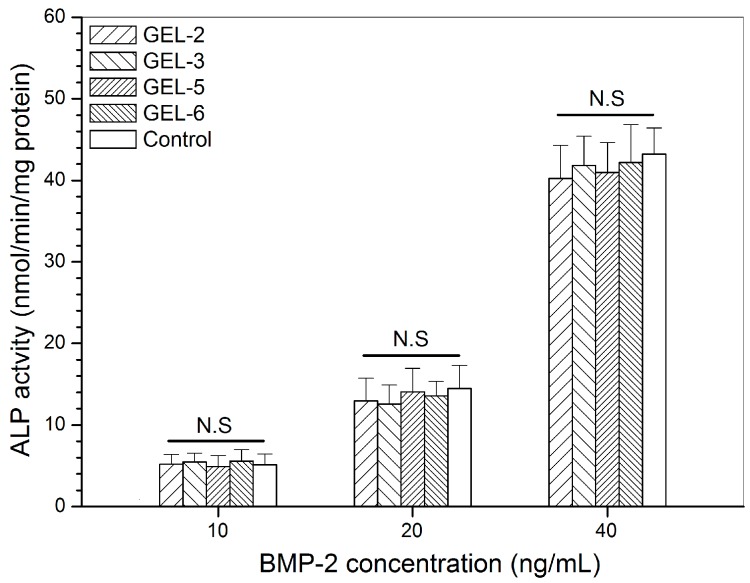
Alkaline phosphatase activity of C2C12 cells that were cultured with BMP-2 released from GEL-3 and GEL-6 gels (see Table 4 for the composition of gels, control: free BMP-2; N.S.: no significance; culture time: 7 days).

It has been reported that BMP-2 is able to reprogram C2C12 cells, a kind of myoblasts, to an osteogenic lineage via BMP-2-involved transdifferentiation [14,15], and thus, BMP-2-induced ALP activity in C2C12 cells can function as an effective indicator for assessing the bioactivity of BMP-2 [28,29]. Four kinds of gels were selected from two sets of gel samples in consideration of their relatively high initial BMP-2 load than others, and the released BMP-2 from them was collected for culturing with C2C12 cells. ALP activity in C2C12 cells was detected and relevant data are depicted in Figure 7. A dose-dependency trend for the BMP-2-induced ALP activity can be seen from Figure 7. There were no significant differences in ALP activity among the detected gels at all applied BMP-2 doses, manifesting that the released BMP-2 and free BMP-2 are almost equally effective in inducing ALP activity in C2C12 cells.

TPP, an ionic crosslinker, has been widely used for crosslinking CH hydrogels, microspheres and NPs in order to endow them with abilities to deliver active biomolecules [18,30]. In the present study, BMP-2-encapsulated HA/CH-PDO NPs were prepared in aqueous media using TPP as a crosslinker and did not involve disadvantageous factors that could denature the bioactivity of protein factors, such as covalent crosslinkers, organic solvents, sonication, and unsuitable processing temperatures or pH values [31,32,33]. Hence, the bioactivity of BMP-2 molecules inside NPs would be well preserved. CH/GP gel has been extensively investigated for diverse biomedical applications, and it shows a well-demonstrated ability for maintaining the bioactivity of the loaded protein factors [10,34]. Thus, the gel matrix would not impair the bioactivity of the loaded BMP-2 molecules. On the basis of above observations, it can be drawn that the presently developed gel system is capable of administering the BMP-2 release at different release rates in a sustained and prolonged manner while effectively retaining the bioactivity of the loaded BMP-2. 

## 4. Conclusions 

Chitosan(CH)-poly(dioxanone)(PDO) copolymers with free amino groups at their CH backbone and soluble character in aqueous media could be combined with polyanionic hyaluronic acid (HA) for preparing HA/CH-PDO polyelectrolyte complex nanoparticles (NPs) with an approximately electrical neutral surface property. Some HA/CH-PDO NPs could encapsulate BMP-2 with high encapsulation efficiency. After embedding such a type of BMP-2-encapsulated HA/CH-PDO NPs into CH/glycerophosphate (GP) hydrogel, the resulting gels could still be able to maintain their thermosensitive phase transition near physiological temperature and pH while showing somewhat reduced but acceptable gelling time. The BMP-2-encapsulated HA/CH-PDO NPs themselves were found to be unsuitable for administering the long-term BMP-2 release due to their severe initial burst release features, whereas the NP-embedded CH/GP gel system was demonstrated to be effective for this goal. Under conditions of optimized compositions for both NPs and gels, the resulting NP-embedded CH/GP gels had the ability to control the BMP-2 release in an approximately linear manner for a few weeks without significantly initial burst release. The release amount of BMP-2 from these NP-embedded gels could possibly be regulated by altering the embedded amount of NPs, which makes it possible to meet varied therapeutic requirements. In addition, this newly developed NP-embedded gel system was capable of well preserving the bioactivity of the loaded BMP-2. 

## Figures and Tables

**Table 1:** Parameters of blank nanoparticles.

Sample Name	HA/CH (m/m)	HA:CH-PDO (m/m) ^b^	Mean Size (nm)	PDI ^c^	ζ (mV)	SI (%)
AN1 ^a^	4:4	-	208.1 ± 8.38	0.13	24.7 ± 3.16	86.4 ± 10.5
AN2	4:3	-	232.6 ± 13.92	0.17	−1.6 ± 0.46	92.9 ± 12.1
AN3	4:2	-	271.8 ± 12.71	0.16	−28.4 ± 3.82	108.2 ± 14.9
AN4	-	4:4	225.3 ± 10.41	0.15	22.4 ± 2.97	89.1 ± 11.6
AN5	-	4:3	248.2 ± 12.16	0.18	−1.9 ± 0.55	95.7 ± 11.8
AN6	-	4:2	297.5 ± 14.73	0.14	−32.2 ± 3.74	104.5 ± 13.2

^a^ These NPs (AN*i*, *i* = 1, 2 and 3) were used as control. ^b^ The PDO content in CH-PDO was 30.2 ± 1.36 wt%. ^c^ PDI: polydispersity index.

**Table 2:** Parameters of BMP-2-encapsulated nanoparticles.

Sample Name	HA/CH (m/m)	HA/CH-PDO ^b^ (m/m)	BMP-2 Feed Amount (ng/mL)	Mean Size (nm)	PDI	ζ (mV)	EE (%)	SI (%)
BN1 ^a^	4:3	-	20	242.1 ± 12.69	0.15	−1.3 ± 0.48	56.2 ± 3.64	97.4 ± 11.7
BN2	4:3	-	40	249.8 ± 10.27	0.18	−1.2 ± 0.37	64.8 ± 3.16	95.2 ± 10.9
BN3	4:3	-	60	254.3 ± 12.08	0.14	−1.4 ± 0.44	68.1 ± 2.93	91.6 ± 12.5
BN4	-	4:3	20	261.5 ± 11.35	0.19	−1.8 ± 0.52	80.4 ± 2.62	101.1 ± 11.8
BN5	-	4:3	40	258.9 ± 12.46	0.17	−1.5 ± 0.46	88.3 ± 3.47	94.8 ± 10.2
BN6	-	4:3	60	267.6 ± 13.14	0.13	−1.1 ± 0.41	89.9 ± 2.85	96.3 ± 12.6

^a^ These NPs (BN*j*, *j* = 1, 2 and 3) were used as control. ^b^ The PDO content in the CH-PDO was 30.2 ± 1.36 wt%.

**Table 3:** Parameters for hydrogels without BMP-2 load.

Sample Name	CH (*w*/*v* %)	GP (*w*/*v* %)	Blank HA/CH NPs (*w*/*v* %)	Blank HA/CH-PDO NPs (*w*/*v* %)	pH	Gelation Time at 37 °C (sec) ^d^	T_i_ (°C) ^e^
CH/GP ^a^	2.0	5.0	-	-	6.97 ± 0.08	550 ± 11	36.5 ± 1.07
GL-I ^b^	2.0	5.0	0.5	-	7.04 ± 0.06	525 ± 10	36.1 ± 1.12
GL-II	2.0	5.0	1.0	-	7.11 ± 0.07	485 ± 10	35.4 ± 1.08
GL-III	2.0	5.0	1.5	-	7.19 ± 0.08	430 ± 11	34.8 ± 1.13
GL-IV ^c^	2.0	5.0	-	0.5	7.08 ± 0.06	540 ± 16	35.7 ± 1.04
GL-V	2.0	5.0	-	1.0	7.07 ± 0.07	495 ± 10	35.2 ± 1.02
GL-VI	2.0	5.0	-	1.5	7.15 ± 0.07	435 ± 10	34.5 ± 1.15

^a^ This gel was used as control. ^b^ These gels (GL-*k*, *k* = I, II, and III) were prepared by using AN2 NPs (see Table 1), and they were used as controls. ^c^ These gels (GL-*l*, *l* = IV, V, and VI) were prepared by using AN5 NPs (see Table 1). ^d^ Gelation time was determined by inverting the vial every 20 s. ^e^ T_i_ indicated the incipient gelation temperature and it was determined via temperature-dependence functions of G′ and G″.

**Table 4:** Parameters for hydrogels containing BMP-2 ^a^.

Sample Name	CH (*w*/*v* %)	BMP-2-Encapsulated HA/CH NPs (*w*/*v* %)	BMP-2-Encapsulated HA/CH-PDO NPs (*w*/*v* %)	BMP-2 Content in Gel (ng/mL)	pH	Gelation Time at 37 °C (sec)	T_i_ (°C)
GEL-1 ^b^	2.0	0.5	-	154.2 ± 9.73	7.06 ± 0.07	530 ± 11	36.3 ± 1.09
GEL-2	2.0	1.0	-	312.6 ± 11.39	7.09 ± 0.08	490 ± 11	35.1 ± 1.16
GEL-3	2.0	1.5	-	472.3 ± 14.62	7.14 ± 0.07	425 ± 10	34.2 ± 1.08
GEL-4 ^c^	2.0	-	0.5	209.5 ± 17.81	7.12 ± 0.09	545 ± 19	35.8 ± 1.15
GEL-5	2.0	-	1.0	421.7 ± 18.95	7.09 ± 0.07	490 ± 11	34.9 ± 1.12
GEL-6	2.0	-	1.5	630.1 ± 19.48	7.11 ± 0.08	420 ± 16	34.4 ± 1.03

^a^ GP content in all gels was 5.0 (*w*/*v*%). ^b^ These gels (GEL-*m*, *m* = 1, 2, and 3) were prepared by incorporating BN2 NPs into CH/GP gel (see Table 2). ^c^ These gels (GEL-*n*, *n* = 4, 5, and 6) were prepared by incorporating BN5 NPs into CH/GP gel (see Table 2).

## Supplementary Materials

The following are available online at https://www.mdpi.com/1999-4923/11/5/214/s1, Figure S1: FTIR spectra for chitosan, PDO and CH-PDO; and a representative. ^1^H NMR spectrum for CH-PDO. Figure S2: SEM images of blank HA/CH NPs (**A**) and blank HA/CH-PDO NPs (**B**). Figure S3: SEM images of BMP-2-encapsulated HA/CH NPs (**A**) and BMP-2-encapsulated HA/CH-PDO NPs (**B**).
