# Supplementary material for: Chitosan-Based Hydrogels Embedded with Hyaluronic Acid Complex Nanoparticles for Controlled Delivery of Bone Morphogenetic Protein-2
Source: Pharmaceutics. 2019 May 4;11(5):214. doi: 10.3390/pharmaceutics11050214 (PMC6572415; doi:10.3390/pharmaceutics11050214)
Supplement: Supplementary file 1 [file pharmaceutics-11-00214-s001.pdf]

pharmaceutics

Supplementary Materials: Chitosan-Based Hydrogels Embedded with Hyaluronic Acid Complex Nanoparticles for Controlled Delivery of Bone Morphogenetic Protein-2

Qing Min, Xiaofeng Yu, Jiaoyan Liu, Jiliang Wu and Ying Wan

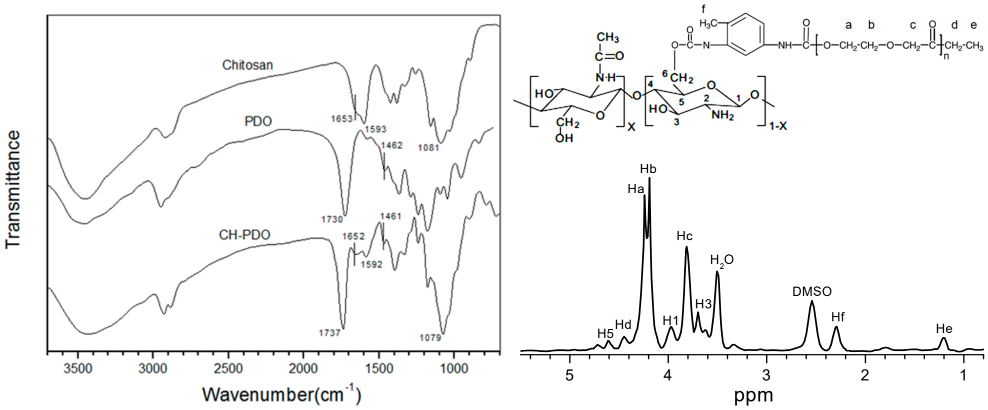

Figure 1. FTIR spectra for chitosan, PDO and CH-PDO; and a representative ^{1}H NMR spectrum for CH-PDO.

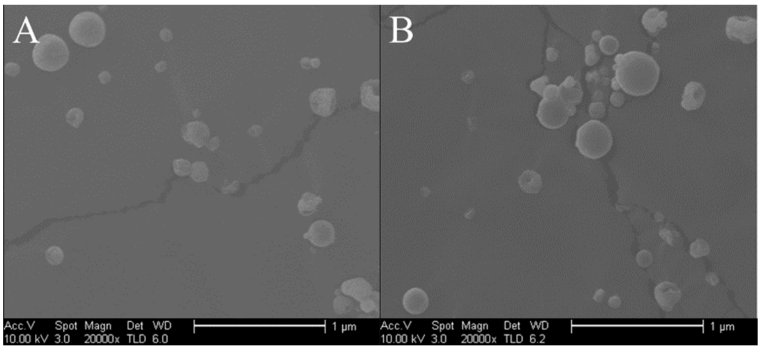

Figure 2. SEM images of blank HA/CH NPs (**A**) and blank HA/CH-PDO NPs (**B**).

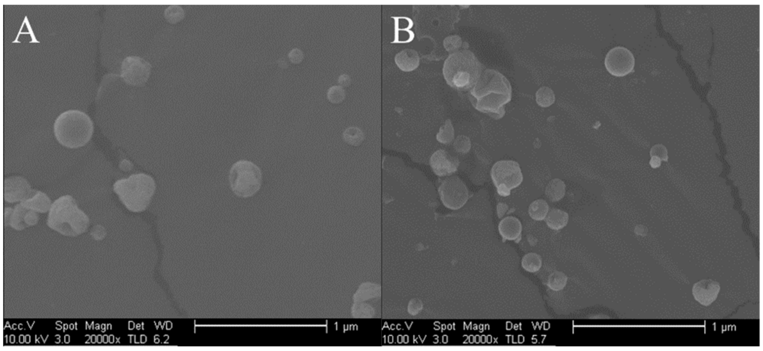

Figure 3. SEM images of BMP-2-encapsulated HA/CH NPs (**A**) and BMP-2-encapsulated HA/CH-PDO NPs (**B**).

© 2019 by the authors. Submitted for possible open access publication under the terms and conditions of the Creative Commons Attribution (CC BY) license (http://creativecommons.org/licenses/by/4.0/).
